# TDNN-Based Ankle Angle Prediction from Lower-Limb Kinematics and Surface EMG

**DOI:** 10.3390/bioengineering13090968

**Published:** 2026-08-24

**Authors:** Abu-Alim Ayazbay, Kassymbek Ozhikenov, Bigaliyeva Zhanar, Sayat Akhmejanov, Nursultan Zhetenbayev, Yerkebulan Nurgizat, Aidos Sultan, Uzbekbayev Arman, Gani Sergazin

**Affiliations:** 1 Department of Robotics and Technical Means of Automation, Satbayev University, Almaty 050013, Kazakhstan; a.ayazbay@aues.kz (A.-A.A.); k.ozhikenov@satbayev.university (K.O.); zh.bigaliyeva@satbayev.university (B.Z.); akhmejanov.s@stud.satbayev.university (S.A.); 2 Department of Aerospace and Electronic Engineering, Almaty University of Power Engineering and Telecommunications, Almaty 050013, Kazakhstan; y.nurgizat@aues.kz (Y.N.); a.sultan@aues.kz (A.S.); a.uzbekbayev@aues.kz (U.A.); 3 Department of Science and Innovations, Mukhametzhan Tynyshbayev ALT University, Almaty 050013, Kazakhstan; g.balbayev@alt.edu.kz

**Keywords:** ankle prosthesis, ankle angle prediction, time delay neural network, surface EMG, lower-limb kinematics, gait analysis

## Abstract

This study presents a Time Delay Neural Network (TDNN) framework for predicting sagittal-plane ankle angle from proximal lower-limb kinematics and surface electromyography (sEMG), with the long-term objective of supporting active ankle prosthesis control. Two TDNN configurations were evaluated: a kinematics-based model (TDNN-Kin) using hip and knee kinematics together with gait-phase information, and an extended model (TDNN-Kin-EMG) incorporating three additional sEMG channels. The models were evaluated using independently held-out treadmill-walking trials from nine able-bodied participants obtained from a publicly available biomechanics dataset. The TDNN-Kin model achieved a mean RMSE of 3.52 ± 0.55° with a mean Pearson correlation coefficient of 0.9338. Incorporating sEMG reduced the mean RMSE to 3.04 ± 0.44°, corresponding to a 13.8% improvement, while the mean correlation coefficient increased to 0.9419. RMSE improved in eight of the nine participants, indicating that muscle-activation information provides complementary information beyond proximal-joint kinematics alone. These findings demonstrate the feasibility of accurate offline ankle-angle prediction using wearable kinematic and physiological signals and provide a foundation for the future integration of data-driven trajectory generation into active ankle prostheses.

## 1. Introduction

Lower-limb amputation substantially alters natural locomotor patterns and can lead to persistent changes in gait biomechanics. Restoring functional and physiologically appropriate walking is therefore one of the main objectives of rehabilitation and prosthetic intervention. Quantitative gait assessment is important in this context because spatiotemporal, kinematic, and kinetic parameters provide objective information on locomotor performance and support the evaluation and adjustment of prosthetic devices [1,2,3]. Recent biomechanical studies have also emphasized the importance of body-weight distribution, gait symmetry, propulsion, and lower-body kinematics when assessing the functional performance of individuals with lower-limb amputation [4].

Despite advances in prosthetic technology, gait asymmetry remains common among individuals with unilateral lower-limb amputation. Compared with the relatively balanced loading observed in able-bodied individuals, amputees often rely more heavily on the intact limb. In the early stages of rehabilitation, individuals with transtibial amputation may support up to 60% of their body weight on the non-amputated limb [5]. Such compensation is also reflected in temporal and kinematic gait asymmetries, with larger deviations generally reported in transfemoral than in transtibial amputees [3,6]. Persistent asymmetry can increase the mechanical demand on the intact limb and may contribute to secondary musculoskeletal problems over time [7,8]. Improving gait symmetry and restoring more physiological lower-limb motion therefore remain important goals in prosthetic development.

The consequences of amputation are not limited to the missing joint. Changes in cadence, stride length, gait symmetry, and pelvic kinematics have been observed, with more proximal amputations generally producing larger deviations [4]. Altered pelvic tilt and obliquity further indicate that compensation occurs across several body segments rather than at a single joint. This coordinated response is particularly relevant to prosthesis control because it suggests that information from the remaining proximal joints may provide useful information about distal limb motion.

The ankle itself has a central role throughout the gait cycle. During early stance, controlled dorsiflexion contributes to knee stabilization, while plantarflexion in late stance supports forward progression and push-off. During swing, sufficient dorsiflexion is required for foot clearance [9,10]. Although the anatomical plantarflexion–dorsiflexion range of the healthy ankle is considerably larger, approximately 30° is typically used during level walking [11,12,13]. Restricting this motion reduces the mechanical contribution of the ankle; experimental evidence has shown that a reduction in ankle range of motion decreases positive ankle work and, when the restriction becomes substantial, alters lower-limb kinematics, joint dynamics, and muscle activation [14]. Reproducing an appropriate ankle trajectory is therefore an important requirement for prosthetic systems intended to support functional gait.

Conventional passive-elastic ankle–foot prostheses, including energy-storage-and-return (ESAR) devices, partially reproduce ankle function by storing and returning elastic energy during stance. Their mechanical output, however, remains below that of the biological ankle. During level walking, ESAR devices have been reported to release less than half of the mechanical energy and less than one-eighth of the peak mechanical power normally generated by the plantarflexor muscles [15]. More importantly, passive devices cannot generate net positive work or actively regulate plantarflexion and dorsiflexion in response to changing gait requirements [15,16]. Users may compensate for this reduced ankle function through increased activity at proximal joints, including greater hip-extensor and knee-flexor recruitment and increased knee muscle co-activation [16,17].

Powered ankle–foot prostheses were introduced to address some of these limitations by actively supplying mechanical work during gait. The BiOM prosthesis, for example, has been reported to provide 57–63% more push-off work during step-to-step transitions than ESAR devices [18,19]. Powered assistance has also been associated with reductions in metabolic demand during level and uphill walking [18,19,20]. However, these benefits are not uniform across walking conditions, indicating that actuator capacity alone is insufficient; the prosthesis must also determine when and how ankle motion or assistance should be delivered.

Several actuation technologies have consequently been explored for powered ankle prostheses. Electric drives are widely used because compact motors can be combined with high-ratio transmission mechanisms [21]. Gabert et al. developed a lightweight powered polycentric ankle–foot prosthesis using a motor-driven ball-screw and gear transmission [22]. Pneumatic systems have also been investigated because of their compliant behavior [23], while electro-hydraulic designs can provide high force and torque with relatively high-power density [24,25]. Each approach involves compromises in weight, transmission efficiency, controllability, and passive resistance. In particular, achieving sufficient active torque while maintaining low resistance during phases in which active assistance is unnecessary remains an important design consideration [26]. Thus, mechanical design and control strategy must be considered together when developing an active ankle prosthesis.

This has led to increasing attention to adaptive and sensor-based control. Karakasis et al. developed an admittance-based controller that adjusted ankle quasi-stiffness according to terrain compliance and reported improved local dynamic stability compared with conventional phase-variable control on compliant surfaces [27]. Physiological sensing provides another route to user-dependent control. Zabre-Gonzalez et al. used surface electromyography (sEMG) within a nonlinear autoregressive model to continuously predict future ankle angle and moment during level walking, stair ascent and descent, and transitions between these activities [28]. These studies demonstrate the value of incorporating information about the user and walking condition rather than relying solely on predefined ankle trajectories.

An alternative source of information is the coordinated motion of the remaining lower-limb joints. Hip, knee, and ankle movements are mechanically and temporally related during gait, and these relationships change systematically with walking conditions. Kwon et al. reported increasing knee flexion and ankle plantarflexion with increasing walking speed and described the relationship between knee and ankle motion as a coupling mechanism [29]. Ankle plantarflexion is also associated with step length and forward progression [29]. Gait speed also influences character-istic lower-limb kinematic and kinetic parameters [30,31]. Taken together, these findings provide a biomechanical basis for examining whether proximal-joint kinematics can be used to estimate distal ankle motion rather than treat-ing ankle motion as an isolated variable.

Recent machine-learning approaches make this possibility increasingly practical. Wearable sensor data can be used to estimate lower-limb joint kinematics without relying on a complete laboratory motion-capture system. Ackland et al. developed a deep-learning framework for real-time estimation of hip, knee, and ankle angles from raw inertial measurement unit (IMU) signals during walking and running [32]. During walking, appropriately positioned individual sensors produced sagittal-plane joint-angle estimates with RMS errors below 4°, while multi-sensor configurations improved simultaneous reconstruction of several joint trajectories [32]. Their results also showed that prediction performance depends on sensor placement and gait speed, indicating that the choice of input information can be as important as the number of sensors.

Physiological information may provide complementary information that is not fully represented by kinematics alone. sEMG reflects muscle activation preceding and accompanying joint movement and has therefore been increasingly incorporated into intention recognition and adaptive assistance. Zhao et al. used calf-muscle sEMG to adapt impedance-control parameters and generate assistive ankle torque in a powered ankle exoskeleton [33]. Together with EMG-based ankle prediction approaches [28], such work suggests that combining mechanical and physiological information may improve the ability of a controller to represent the user’s intended movement. At the same time, whether sEMG provides a meaningful improvement over proximal-joint kinematics alone for ankle trajectory prediction remains an important practical question, particularly when sensor complexity is considered.

The present work builds on two previous stages of our active ankle–foot prosthesis project. The first stage established the mechanical architecture of a 2-DoF ankle–foot prosthesis and included parametric CAD development, kinematic and dynamic modeling, and preliminary motion analysis [34]. The subsequent stage resulted in an improved microprocessor-controlled prototype incorporating an SFU1204-300 ball-screw transmission, NEMA 17 stepper motor, ESP32-based control system, MPU9250 IMU, homing mechanism, and wireless control interface [35]. Laboratory evaluation demonstrated repeatable dorsiflexion–plantarflexion motion over an approximately 39–40° sagittal range, with positional repeatability within ±0.3–0.5° [35]. These studies established the mechanical, actuation, sensing, and embedded-control platform; the next step is to determine how an appropriate ankle trajectory can be generated from information available from the user during gait.

Accordingly, the present study investigates a data-driven approach for predicting sagittal-plane ankle angle from proximal lower-limb information, with the long-term objective of providing reference trajectories for active ankle prosthesis control. Two time-delay neural network (TDNN) models are evaluated under identical training and testing conditions. The first uses hip angle, knee angle, and gait-phase information, while the second additionally incorporates three sEMG channels. This design enables a direct assessment of whether the inclusion of sEMG provides a meaningful improvement in ankle-angle prediction beyond proximal-joint kinematics alone.

The main contributions of this study are: (1) the development of a TDNN-based framework for predicting ankle angle from proximal-joint kinematics and gait-phase information; (2) a direct comparison between kinematics-only and kinematics-plus-sEMG models under the same experimental conditions; and (3) subject-wise evaluation using held-out gait trials to assess prediction accuracy and the consistency of the contribution provided by sEMG. The resulting framework represents an offline proof-of-concept and an intermediate step toward the future integration of data-driven trajectory generation into an active ankle-foot prosthesis platform.

## 2. Active Ankle Prosthesis and Proposed Prediction Framework

### 2.1. Active Ankle Prosthesis Prototype

**Figure 1:**
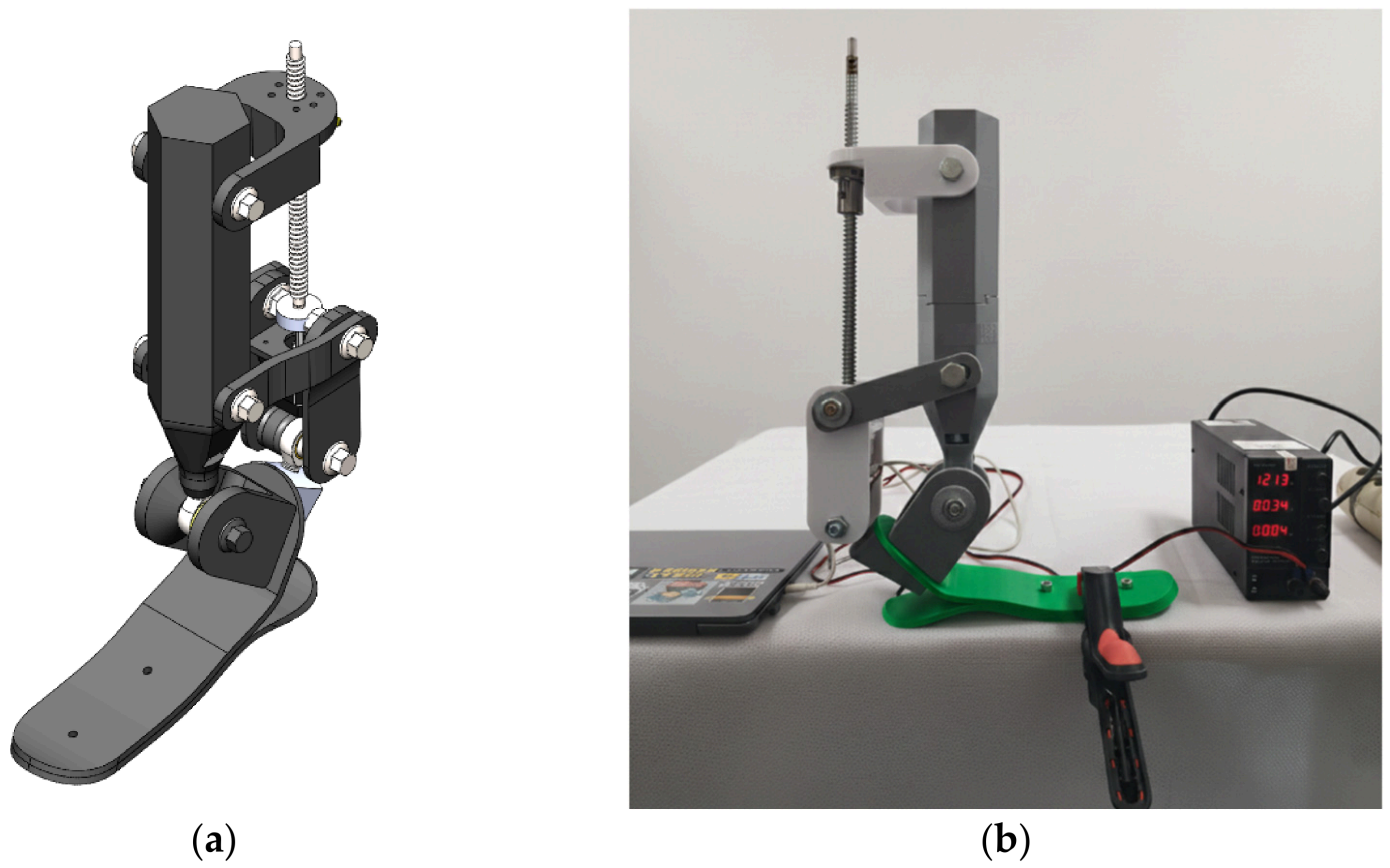
Active ankle–foot prosthesis prototype: (**a**) CAD model of the mechanical architecture; (**b**) fabricated prototype during laboratory testing.

The active ankle–foot prosthesis used as the target platform in this study was developed through successive mechanical and experimental stages described in our previous works [34,35]. The current prototype consists of a rigid lower-leg frame, an articulated ankle–foot assembly, and a motor-driven linear transmission mechanism. The CAD model and the fabricated laboratory prototype are shown in Figure 1a,b, respectively.

The ankle mechanism converts the linear displacement of the screw-driven actuator into rotational motion of the foot through a system of rigid links and revolute joints (Figure 1a). The main load-bearing frame supports the actuation mechanism, while the lower linkage transfers actuator displacement to the ankle joint. This arrangement enables controlled dorsiflexion and plantarflexion without placing the actuator directly at the ankle axis. The foot structure provides the mechanical interface for transmitting the generated ankle motion to the prosthetic foot.

A physical prototype was manufactured to verify the operation of the proposed mechanism under laboratory conditions (Figure 1b). The fabricated system preserves the principal kinematic arrangement of the CAD design, including the vertical linear drive, connecting linkage, ankle joint, and foot structure. During preliminary bench testing, the prototype demonstrated repeatable dorsiflexion–plantarflexion motion over an approximately 39–40° sagittal-plane range [35]. The developed prototype therefore provides the experimental hardware platform used to investigate the proposed ankle trajectory prediction framework.

### 2.2. Actuation, Sensing, and Embedded Control System

**Figure 2:**
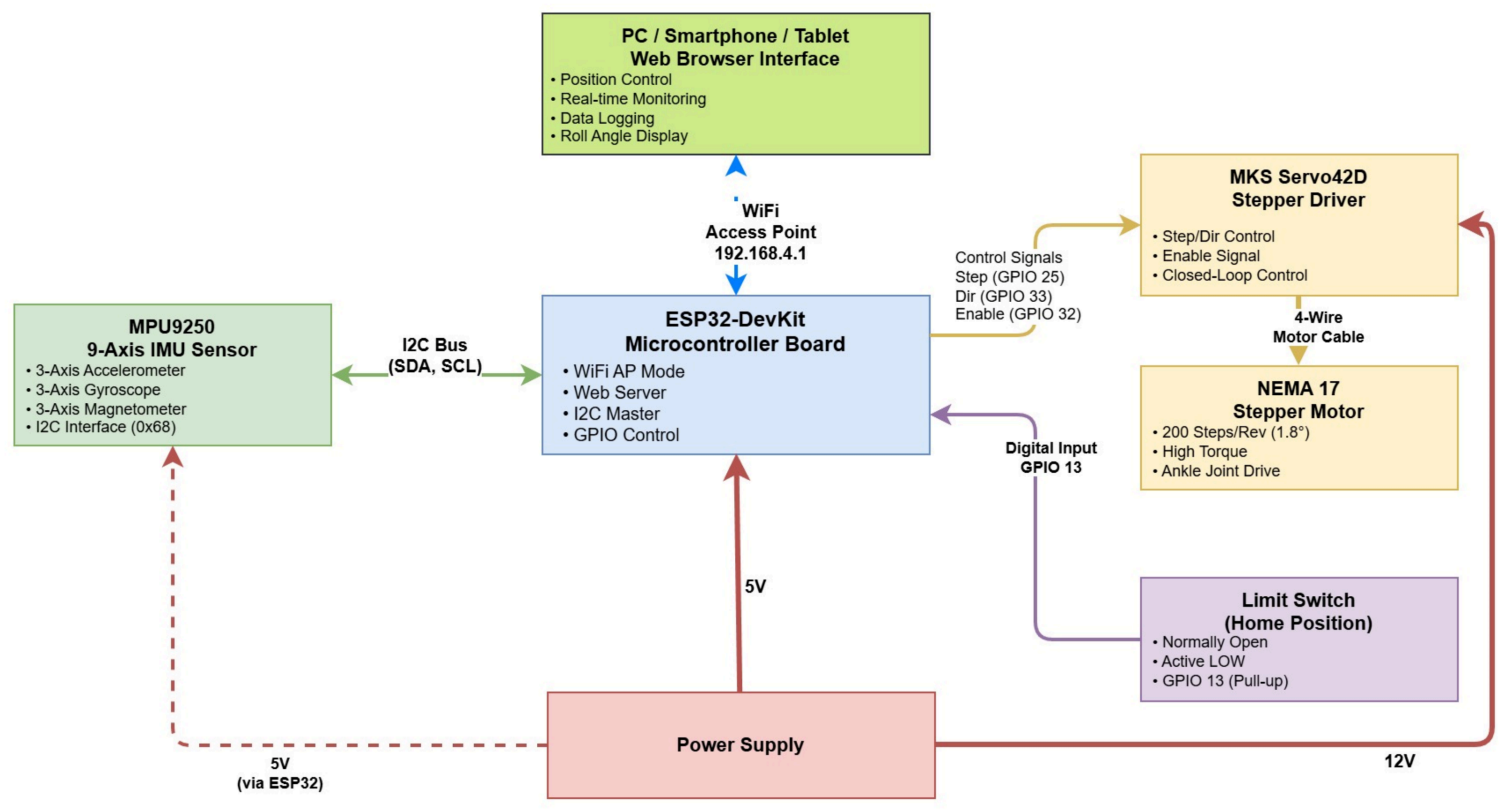
Actuation, sensing, and embedded control architecture of the active ankle–foot prosthesis.

The actuation and embedded control architecture of the developed ankle prosthesis is shown in Figure 2. The system is built around an ESP32-DevKit microcontroller, which coordinates motor actuation, sensor acquisition, homing, and communication with the user interface. An MKS Servo42D driver receives step, direction, and enable signals from the ESP32 and controls the NEMA 17 stepper motor. The rotary motion of the motor is transmitted through the ball-screw mechanism described in Section 2.1, producing linear displacement of the linkage and the corresponding dorsiflexion or plantarflexion of the ankle joint.

An MPU9250 nine-axis inertial measurement unit (IMU) is connected to the ESP32 through the I^2^C interface and provides three-axis acceleration, angular velocity, and magnetic-field measurements. In the developed prototype, the IMU is used to monitor the orientation of the prosthetic segment and provide angular information during operation. A normally open limit switch connected to GPIO 13 defines the mechanical home position and provides a repeatable reference for initialization before motion commands are executed.

The ESP32 also operates as a WiFi access point and hosts a browser-based interface that can be accessed from a computer, smartphone, or tablet. The interface provides position commands, real-time monitoring, data logging, and roll-angle visualization. The control electronics and sensors operate from the 5 V supply provided through the ESP32, while the motor drive is supplied separately at 12 V, as indicated in Figure 2. This architecture provides the embedded hardware interface required for future integration of the predicted ankle-angle trajectory with the prosthesis actuator.

### 2.3. Proposed TDNN-Based Ankle Trajectory Prediction Framework

**Figure 3:**
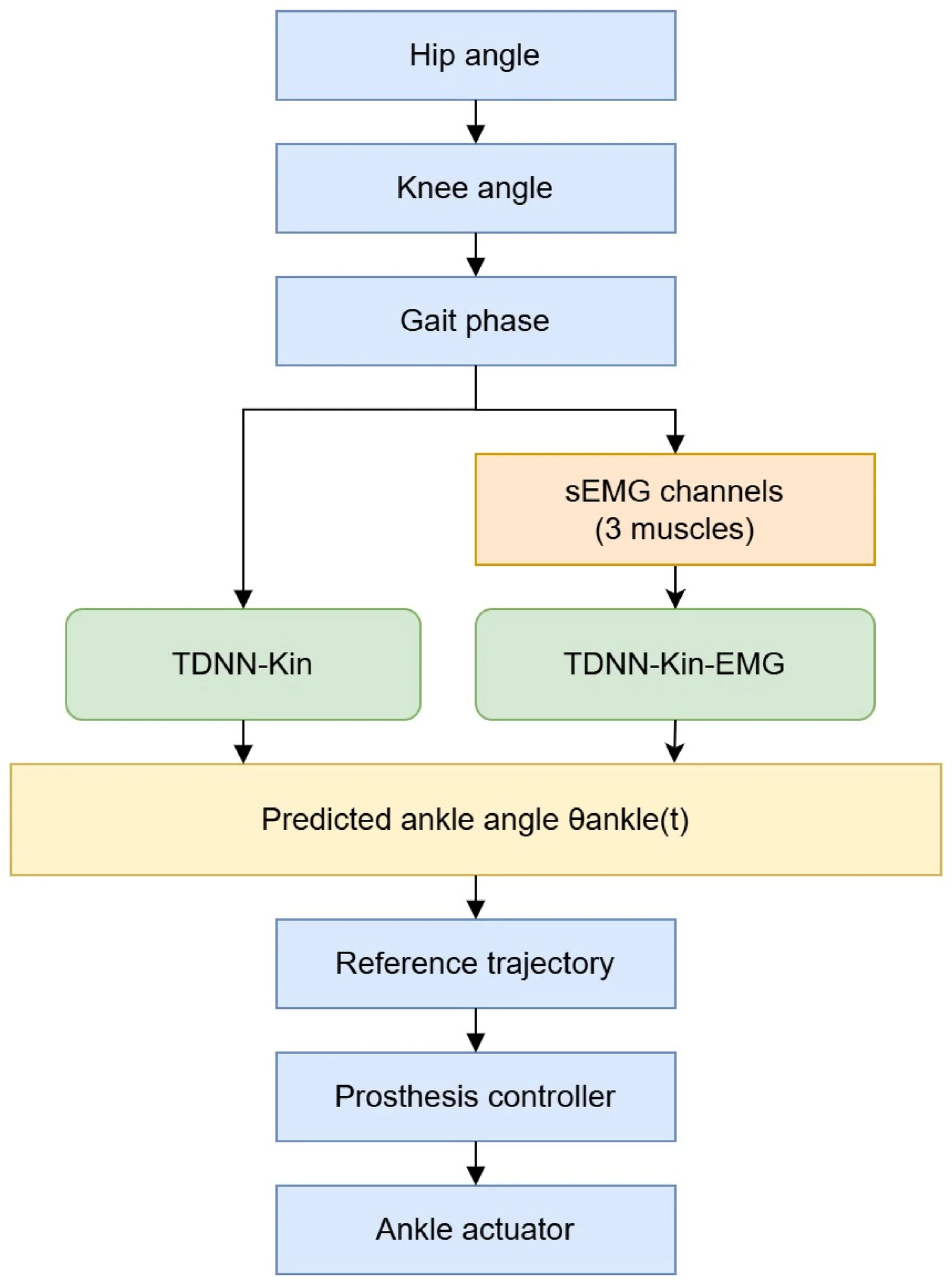
Proposed TDNN-based ankle trajectory prediction framework for future integration into active ankle prosthesis control.

The proposed prediction framework was developed to generate a sagittal-plane ankle-angle trajectory from lower-limb information available during gait, as indicated in Figure 3. Rather than prescribing a fixed ankle trajectory, the framework estimates the ankle angle from the motion of the proximal joints and the current gait phase. A time-delay neural network (TDNN) was selected because ankle motion depends not only on the instantaneous state of the lower limb but also on its recent temporal history.

Two input configurations were considered. The first model, denoted as TDNN-Kin, uses hip angle, knee angle, and gait-phase information as inputs. This configuration was designed to determine whether proximal-joint kinematics alone provide sufficient information for reconstruction of the ankle trajectory. The second model, TDNN-Kin-EMG, uses the same kinematic and gait-phase inputs together with three sEMG channels. The additional physiological signals were included to evaluate whether muscle activation provides complementary information that improves ankle-angle prediction beyond that obtained from kinematics alone.

For both configurations, the input signals are organized into a temporal window containing the current and preceding samples. The TDNN processes this time-delayed input sequence and produces an offline estimate of the ankle-angle trajectory. The predicted ankle trajectory is intended as a reference trajectory for future integration into the low-level position controller of the active ankle prosthesis.

A temporal window of 20 samples (100 ms at 200 Hz) was selected because it provides sufficient recent motion history while maintaining a short prediction latency suitable for future real-time implementation. Preliminary experiments indicated that larger windows produced only marginal improvements while increasing computational cost.

The two models are evaluated using the same dataset, preprocessing procedure, temporal window, network architecture, and training protocol. Consequently, the difference in prediction performance between TDNN-Kin and TDNN-Kin-EMG can be attributed to the inclusion of sEMG information rather than to differences in model configuration. The detailed dataset preparation, TDNN architecture, training procedure, and evaluation metrics are presented in Section 3.

### 2.4. Integration Concept for Prosthesis Control

The predicted ankle angle is considered as a potential reference signal for future implementation in the prosthesis position-control system. The predicted ankle angle, ${\hat{\theta }}_{ankle}(t)$, is considered as a reference signal for the prosthesis position-control system. In the intended configuration, hip and knee kinematics and gait-phase information are processed by the TDNN to generate a continuous ankle-angle trajectory. When available, sEMG signals can be included as additional inputs according to the TDNN-Kin-EMG configuration described in Section 2.3.

The predicted ankle trajectory is then converted into the corresponding actuator position command based on the kinematic relationship between the linear ball-screw displacement and ankle rotation. The ESP32-based controller provides the interface between the reference command and the motor drive, while the IMU mounted on the prosthesis provides angular information that can be used to monitor the resulting ankle motion. The limit switch establishes a repeatable home position before actuation. In this way, the prediction layer and the existing embedded control system could be integrated without changing the basic mechanical architecture of the prosthesis.

The present study focuses on the development and offline validation of the ankle-angle prediction model. The TDNN output has not yet been implemented as a real-time command on the physical prosthesis. Therefore, the proposed architecture represents an integration concept for the next stage of the project rather than a validated closed-loop prosthesis controller. Future implementation will require real-time signal acquisition, execution of the trained TDNN model on the control platform, conversion of the predicted ankle angle into actuator displacement, and experimental validation under loaded walking conditions.

In addition, the present work does not evaluate closed-loop performance in human subjects, and the proposed control strategy therefore remains to be validated under real-time experimental conditions.

## 3. Materials and Methods

### 3.1. Dataset and Participants

This study used the publicly available lower-limb biomechanics dataset reported by Camargo et al. [12]. The complete dataset contains synchronized motion-capture, biomechanical, and surface electromyography (sEMG) recordings from 22 able-bodied participants performing a range of locomotor tasks, including level-ground walking, treadmill walking, stair negotiation, and ramp locomotion. For the present analysis, data from nine participants (AB06–AB14) recorded during treadmill walking at multiple speeds were selected.

Each participant contributed 15 treadmill walking trials. One trial (treadmill_07_01) from each participant was reserved exclusively for the final evaluation, while the remaining 14 trials per participant (126 trials in total) were used for model development. Kinematic and gait-event data were sampled at 200 Hz, whereas raw sEMG signals were recorded at 1000 Hz. The dataset provides inverse-kinematics (IK) estimates of lower-limb joint angles, ground-contact (GC) information for identifying gait events and determining gait-cycle phase, and sEMG recordings from eleven lower-limb and trunk muscles [12]. In the present study, hip and knee joint angles were used as proximal kinematic inputs, while the ankle angle served as the prediction target. Three sEMG channels were additionally selected for the model incorporating physiological information, as described in Section 3.3.

The demographic characteristics of the nine participants included in the analysis are summarized in Table 1. All participants provided informed consent as part of the original data-collection protocol [12]. No new participant recruitment or experimental data collection was performed for the present study.

### 3.2. Input Features and Time-Delay Representation

Two input configurations were defined to evaluate the contribution of sEMG information to ankle-angle prediction. The first configuration, referred to as TDNN-Kin, contained five kinematic and gait-related input channels: hip flexion angle (°), hip angular velocity (°/s), knee flexion angle (°), knee angular velocity (°/s), and gait-cycle phase (%). Hip and knee angular velocities were calculated from the corresponding inverse-kinematics joint-angle signals using central numerical differentiation. The gait-cycle phase was calculated offline using consecutive heel-strike events obtained from the ground-contact annotations provided in the dataset. Because identification of the subsequent heel strike requires future information, the gait phase was available only during offline processing and was not intended for direct real-time implementation.

The second configuration, TDNN-Kin-EMG, contained the same five inputs together with three sEMG channels recorded from the gastrocnemius medialis, tibialis anterior, and soleus muscles, resulting in eight input channels. The original sEMG signals, sampled at 1000 Hz, were rectified and reduced to 200 Hz to match the kinematic data by averaging each group of five consecutive samples. No additional high-pass, low-pass, or notch filtering was applied because the present study relied on the preprocessed signals supplied within the public dataset.

These three muscles were selected because they represent the primary ankle dorsiflexor and plantarflexor muscle groups and provide the most direct physiological information related to sagittal-plane ankle motion.

Before model training, all input variables and the target ankle flexion angle were standardized using z-score normalization. The mean and standard deviation used for normalization were calculated exclusively from the training data and subsequently applied to the corresponding validation and test data. A common normalization scheme was applied across the model-development data to ensure consistent scaling of all input variables. The independent held-out treadmill_07_01 trial from each participant was excluded from the normalization procedure to prevent information leakage. Since data from all participants were used during model development, the evaluation reflects generalization to unseen trials from known participants rather than to unseen individuals.

For the TDNN input, the normalized features were arranged as time-delayed sequences so that each ankle-angle prediction was based on the current input values together with a defined history of preceding samples. The same temporal representation was used for both TDNN-Kin and TDNN-Kin-EMG, ensuring that the two models differed only in the inclusion of the three sEMG channels. The specific delay length and network configuration are described in Section 3.4.

### 3.3. TDNN Architecture

A Time Delay Neural Network (TDNN) was used to predict the sagittal-plane ankle angle from the input signals described in Section 3.3. At each time step t, the network receives the current input sample together with the preceding 19 samples, forming a 20-sample temporal window $\left[t-19,\ldots ,t\right]$. At the sampling frequency of 200 Hz, this window corresponds to 100 ms of input history. The TDNN is implemented as a feedforward network without recurrent connections or output feedback.

The 20-sample window was selected as a compromise between capturing the short-term temporal dependency of gait and maintaining a sufficiently low computational latency for future real-time implementation. Preliminary testing indicated that increasing the window length produced only marginal improvements in prediction accuracy.

The network consists of a single hidden layer with 15 neurons using the hyperbolic tangent (tanh) activation function and a single linear output neuron that estimates the ankle flexion angle, $\hat{y}(t)$. The same hidden-layer and output configuration was used for both input configurations to allow a direct comparison of the effect of adding sEMG information.

For the TDNN-Kin model, five input signals are evaluated over 20 time steps, resulting in an input vector of 5×20=100 elements. The corresponding first-layer weight matrix $W_{1}$ has dimensions 15×100, followed by 15 hidden-layer biases. The output layer contains a 1×15 weight vector $W_{2}$ and a single bias, giving a total of 1531 trainable parameters.

**Figure 4:**
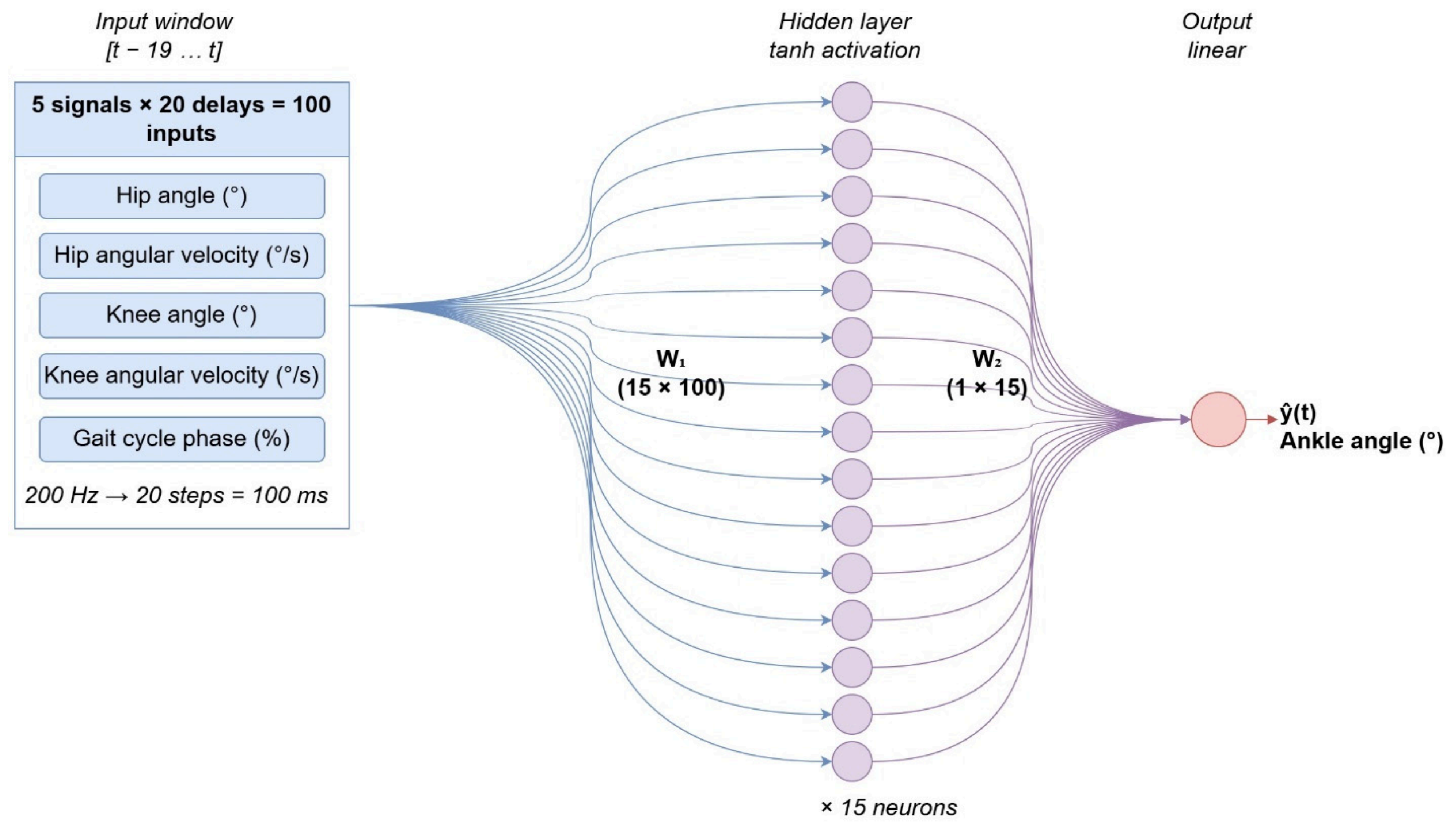
Architecture of the TDNN-Kin model for ankle-angle prediction using a 20-sample (100 ms) temporal input window, 15-neuron tanh hidden layer, and linear output neuron.

For the TDNN-Kin-EMG model, the three sEMG channels are added to the five kinematic and gait-related inputs, increasing the input dimensionality to 8×20=160. The first-layer weight matrix therefore increases to 15×160, while the hidden and output layers remain unchanged. This configuration contains 2431 trainable parameters. The architecture of the TDNN-Kin model is illustrated in Figure 4.

### 3.4. Training Protocol

All treadmill-walking trials from participants AB06–AB14 were treated as independent time-series sequences. For each participant, the treadmill_07_01 trial was reserved as an independent held-out test sequence and was excluded from all stages of model development, including data normalization, network training, validation, internal testing, and hyperparameter selection. Consequently, the held-out trials were accessed only once for the final performance evaluation. The remaining 14 trials from each participant (126 trials in total) were used for model development.

The model-development dataset was prepared at the trial level to preserve the temporal continuity of individual walking sequences. Before training, the order of the development trials was randomized, after which all selected trials were concatenated while maintaining the original sample order within each trial. This procedure reduced any dependence on the original recording order without disrupting the temporal structure of the gait data.

Both TDNN configurations were trained using the Levenberg–Marquardt backpropagation algorithm (trainlm). The concatenated model-development sequence was divided using MATLAB’s (version R2025a) divideblock function, allocating 80% of the samples for training, 10% for validation, and 10% for internal testing. Early stopping was employed based on the validation error, with the maximum number of consecutive validation failures set to 50 (max_fail = 50). L2 regularization with a coefficient of 0.01 was applied to reduce overfitting.

The maximum number of training epochs was set to 1000; however, both TDNN-Kin and TDNN-Kin-EMG reached convergence within approximately 25 epochs. Identical network architecture, optimization settings, stopping criteria, and regularization parameters were used for both models so that any difference in prediction performance could be attributed to the inclusion of the three sEMG channels rather than to differences in the training procedure. All model development and training were performed in MATLAB R2024b using the timedelaynet implementation.

### 3.5. Evaluation Metrics

Each trained model was independently evaluated using the held-out treadmill_07_01 trial from each of the nine participants (AB06–AB14). These trials were excluded from model training, validation, and hyperparameter selection and were used only for the final assessment of prediction performance.

Prediction accuracy was quantified using the root mean square error (RMSE) and Pearson correlation coefficient (R). RMSE, expressed in degrees, was used to quantify the magnitude of the difference between the measured and predicted ankle angles, whereas R was used to assess the agreement in waveform shape and temporal variation between the two signals. Both metrics were calculated separately for each participant to evaluate inter-subject consistency.

Because the TDNN operates as a feedforward model using a fixed temporal input window, evaluation was performed directly on the held-out sequences without recurrent-state initialization or switching between open- and closed-loop network configurations. The same evaluation procedure was applied to the TDNN-Kin and TDNN-Kin-EMG models to allow a direct comparison of their prediction performance.

To compare the two TDNN configurations, paired statistical analyses were performed on the participant-wise RMSE and Pearson correlation values. Normality of the paired differences was assessed before selecting the appropriate statistical test. Depending on the normality assumption, either a paired *t*-test or a Wilcoxon signed-rank test was applied. Statistical significance was evaluated at *p* < 0.05.

To compare the performance of the TDNN-Kin and TDNN-Kin-EMG models across participants, paired statistical analyses were performed using the Wilcoxon signed-rank test. The test was applied separately to the participant-wise RMSE and Pearson correlation coefficient (R) values. Statistical significance was determined at a significance level of *p* < 0.05.

## 4. Results

### 4.1. Training Convergence

**Figure 5:**
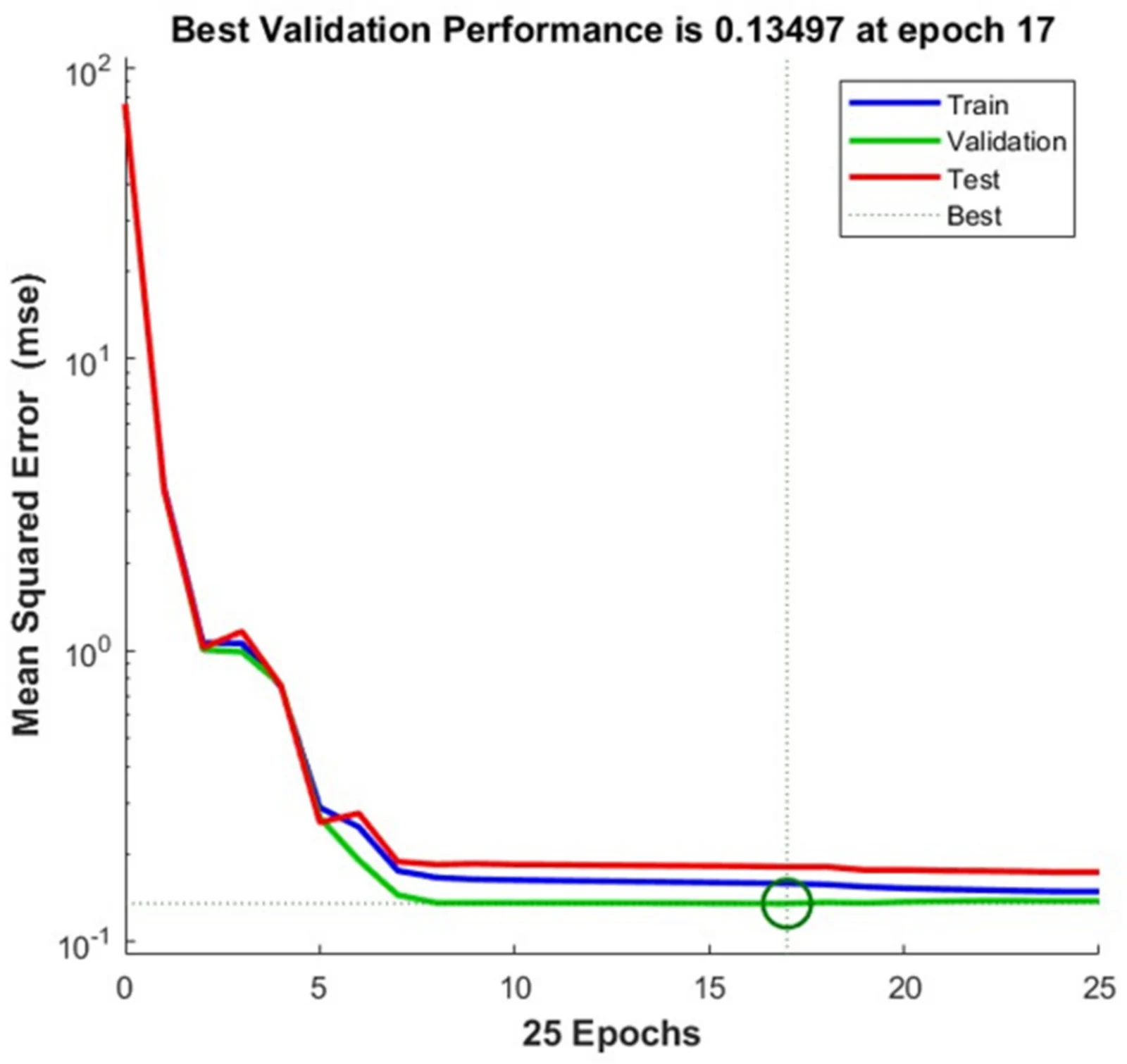
Training convergence of the TDNN-Kin model. The minimum validation MSE of 0.13497 was obtained at epoch 17.

Both TDNN configurations showed rapid convergence during training. As shown in Figure 5, the TDNN-Kin model reached its minimum validation mean squared error (MSE) of 0.13497 at epoch 17. The training process continued to 25 epochs, with only minor changes in performance after the minimum validation error was reached. The training, validation, and internal test curves followed similar trends and remained close after convergence.

**Figure 6:**
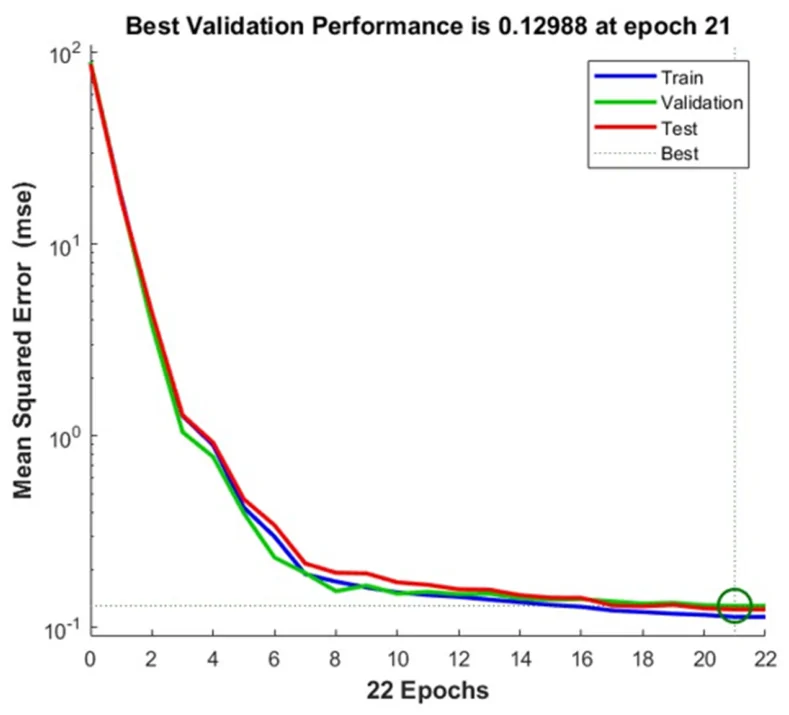
Training convergence of the TDNN-Kin-EMG model. The minimum validation MSE of 0.12988 was obtained at epoch 21.

The TDNN-Kin-EMG model showed a comparable convergence pattern (Figure 6). Its minimum validation MSE was 0.12988 at epoch 21, and training terminated after 22 epochs. The addition of the three sEMG channels therefore resulted in a slightly lower minimum validation MSE than the kinematics-only configuration (0.12988 vs. 0.13497). The training, validation, and internal test curves again remained closely grouped during the final epochs, with no marked divergence between the three subsets.

Overall, both models reached stable validation performance within a relatively small number of training epochs. The convergence results indicate that the selected TDNN architecture was sufficient for both input configurations, while the inclusion of sEMG produced only a modest improvement in validation MSE at the training stage. Prediction performance on the independent held-out test trials is presented in the following sections.

### 4.2. Held-Out Prediction Accuracy

Prediction performance on the independent held-out trial (treadmill_07_01) is summarized in Table 2 for all nine participants. The TDNN-Kin model achieved a mean RMSE of $3.52\pm 0.55^{\circ }$ and a mean Pearson correlation coefficient of R=0.9338. With the addition of the three sEMG channels, the TDNN-Kin-EMG model reduced the mean RMSE to $3.04\pm 0.44^{\circ }$, corresponding to an overall reduction of 13.8%. The mean correlation coefficient also increased from 0.9338 to 0.9419.

The effect of adding sEMG varied across participants. The TDNN-Kin-EMG model reduced RMSE in eight of the nine participants, with improvements ranging from 1.7% for AB11 to 31.1% for AB06. The only exception was AB08, for whom RMSE increased from 3.36° to 3.75°. Improvements exceeding 20% were observed for AB06, AB09, AB10, and AB12. The standard deviation of RMSE across participants also decreased from 0.55° to 0.44° with the inclusion of sEMG.

Changes in waveform correlation were less uniform. Pearson R increased for six participants (AB06, AB07, AB10, AB12, AB13 and AB14). This result illustrates that improvements in absolute prediction error do not necessarily correspond to an increase in waveform correlation.

A paired statistical comparison using the Wilcoxon signed-rank test showed that the inclusion of sEMG significantly reduced the RMSE across the nine participants (median paired difference = 0.39°, *p* = 0.039, effect size r = 0.69, 95% CI = [0.06°, 1.15°]). No statistically significant difference was observed for the Pearson correlation coefficient (median paired difference = 0.005, *p* = 0.359).

### 4.3. Prediction Waveforms

**Figure 7:**
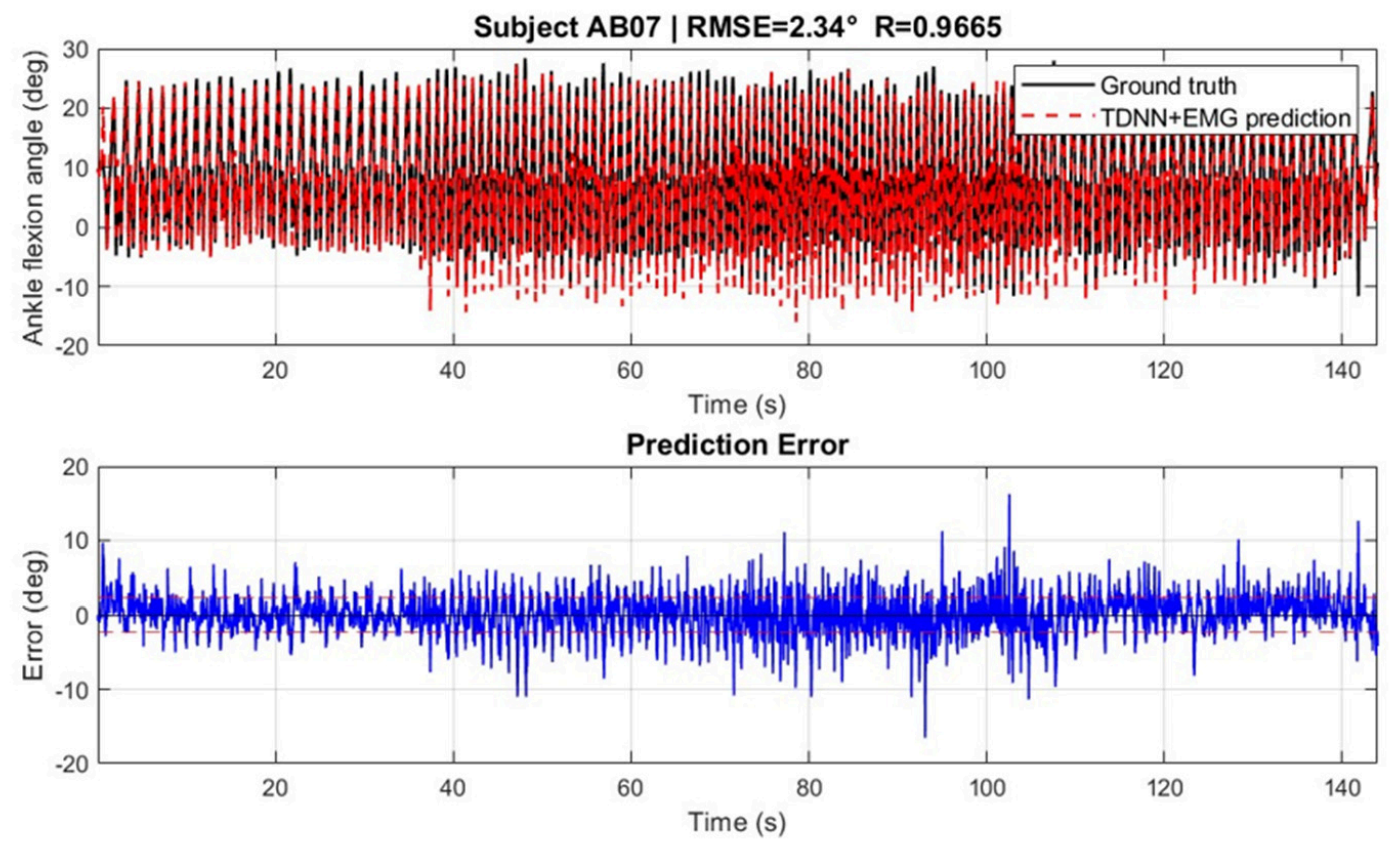
Measured and predicted ankle-angle trajectories for participant AB07 using the TDNN-Kin-EMG model ($RMSE=2.34^{\circ }$, R=0.9665). Upper panel: measured ankle angle (black) and predicted ankle angle (red dashed); lower panel: prediction error over the complete held-out trial.

**Figure 8:**
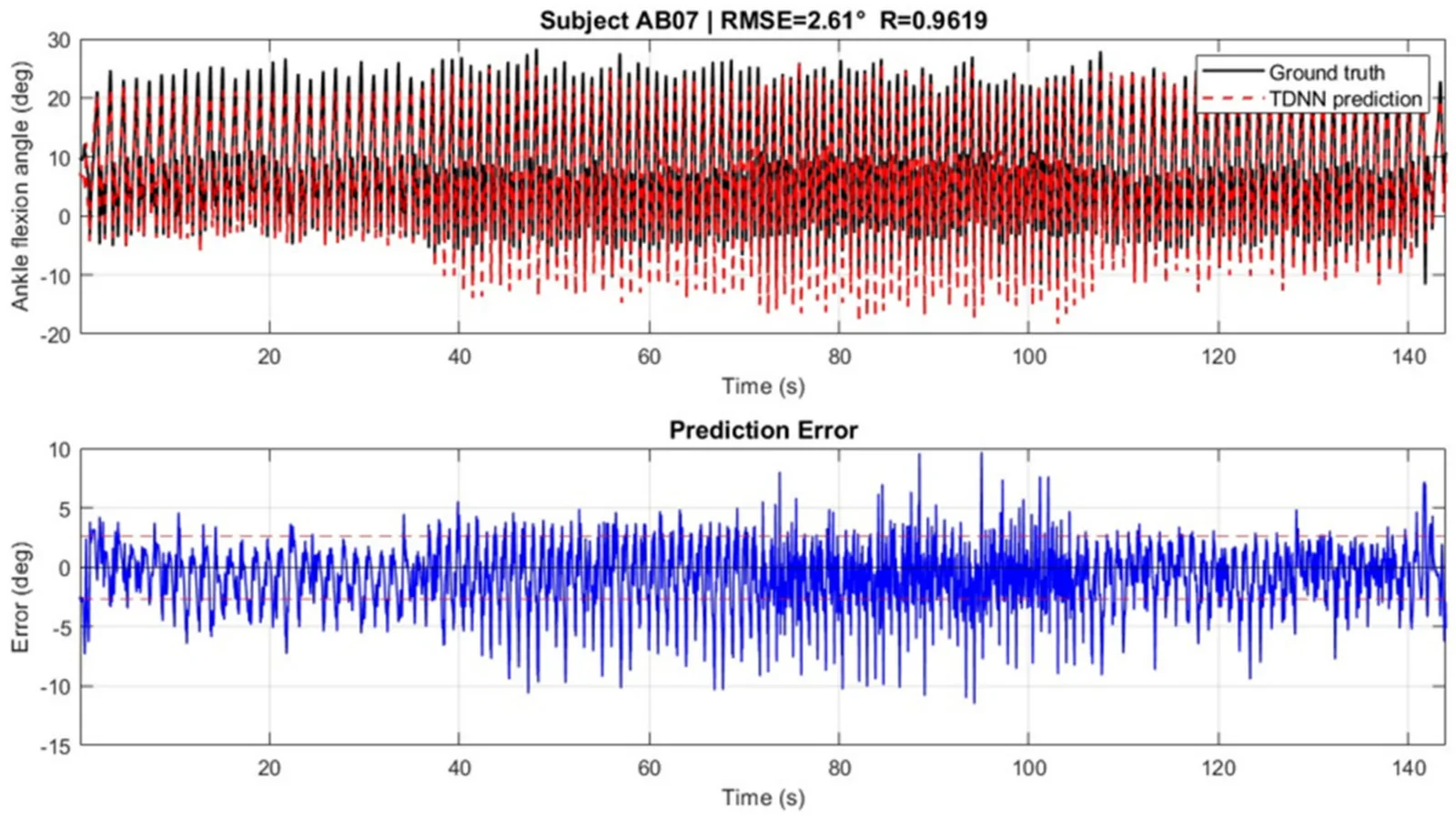
Measured and predicted ankle-angle trajectories for participant AB07 using the TDNN-Kin model ($RMSE=2.61^{\circ }$, R=0.9619). Upper panel: measured ankle angle (black) and predicted ankle angle (red dashed); lower panel: prediction error over the complete held-out trial.

Representative ankle-angle prediction waveforms for participant AB07 are shown in Figure 7 and Figure 8. Both models reproduced the periodic pattern of the measured ankle trajectory throughout the approximately 144 s held-out trial. For the TDNN-Kin-EMG model (Figure 7), the predicted trajectory closely followed the measured ankle angle, resulting in an RMSE of 2.34° and a Pearson correlation coefficient of R=0.9665. These results represent offline prediction on an independent held-out trial and should not be interpreted as real-time prosthesis control performance.

The corresponding prediction error remained centered near zero for most of the trial, although larger transient deviations occurred at several points in the gait sequence. The magnitude of these deviations varied over time, with more pronounced errors observed in portions of the trial exhibiting larger changes in ankle-angle amplitude.

The TDNN-Kin model also reproduced the overall ankle-angle waveform (Figure 8), with an RMSE of 2.61° and R=0.9619. Compared with the kinematics-only configuration, the addition of sEMG reduced the RMSE for AB07 by approximately 10.3% and slightly increased the correlation coefficient. Visual comparison of the error traces also shows that both models maintained prediction errors close to zero over much of the trial, while intermittent deviations remained in both configurations.

### 4.4. Per-Subject Error Distribution

**Figure 9:**
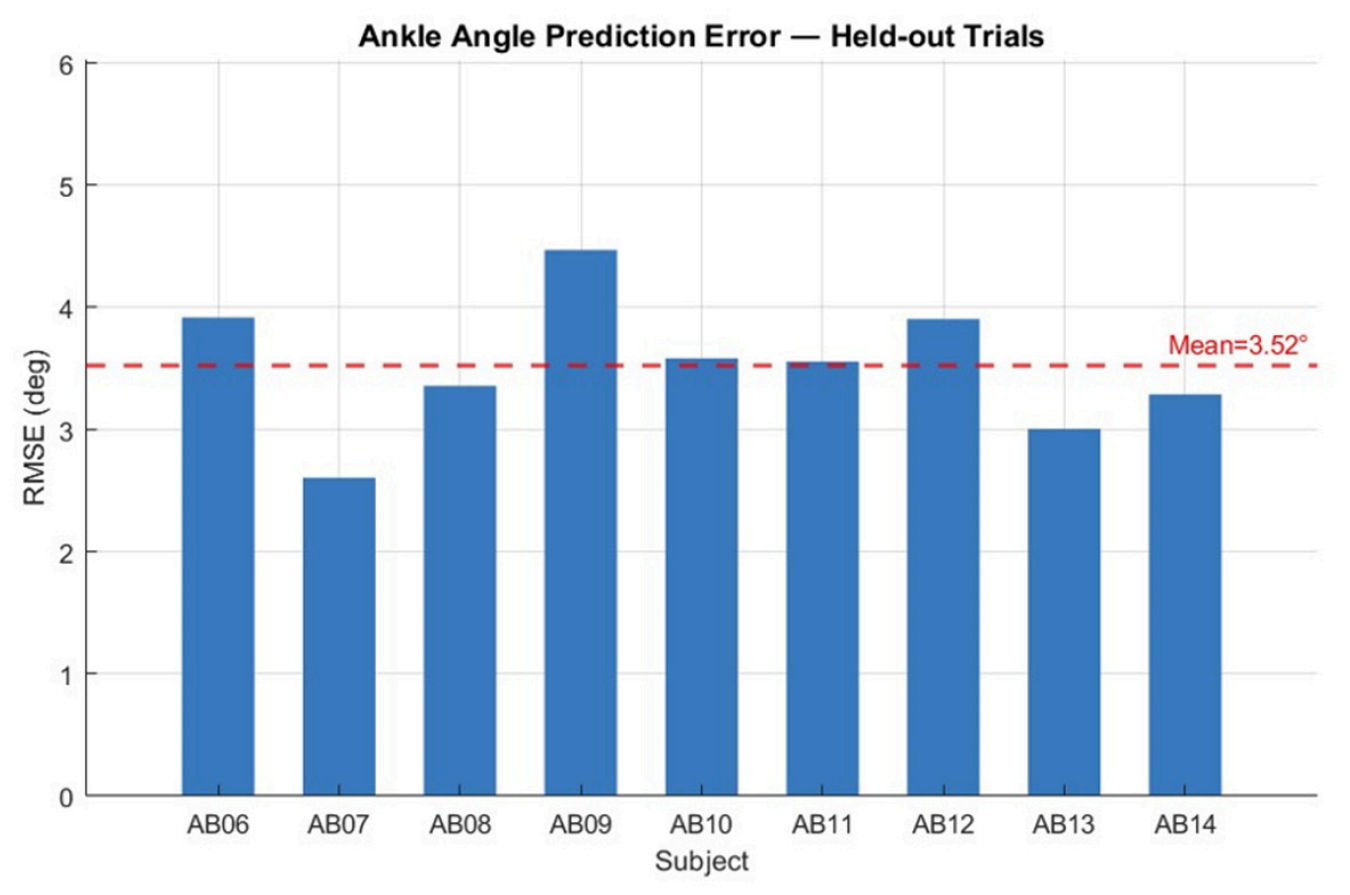
Per-subject RMSE for the TDNN-Kin model on the held-out trials. The dashed line indicates the across-subject mean RMSE of 3.52°.

**Figure 10:**
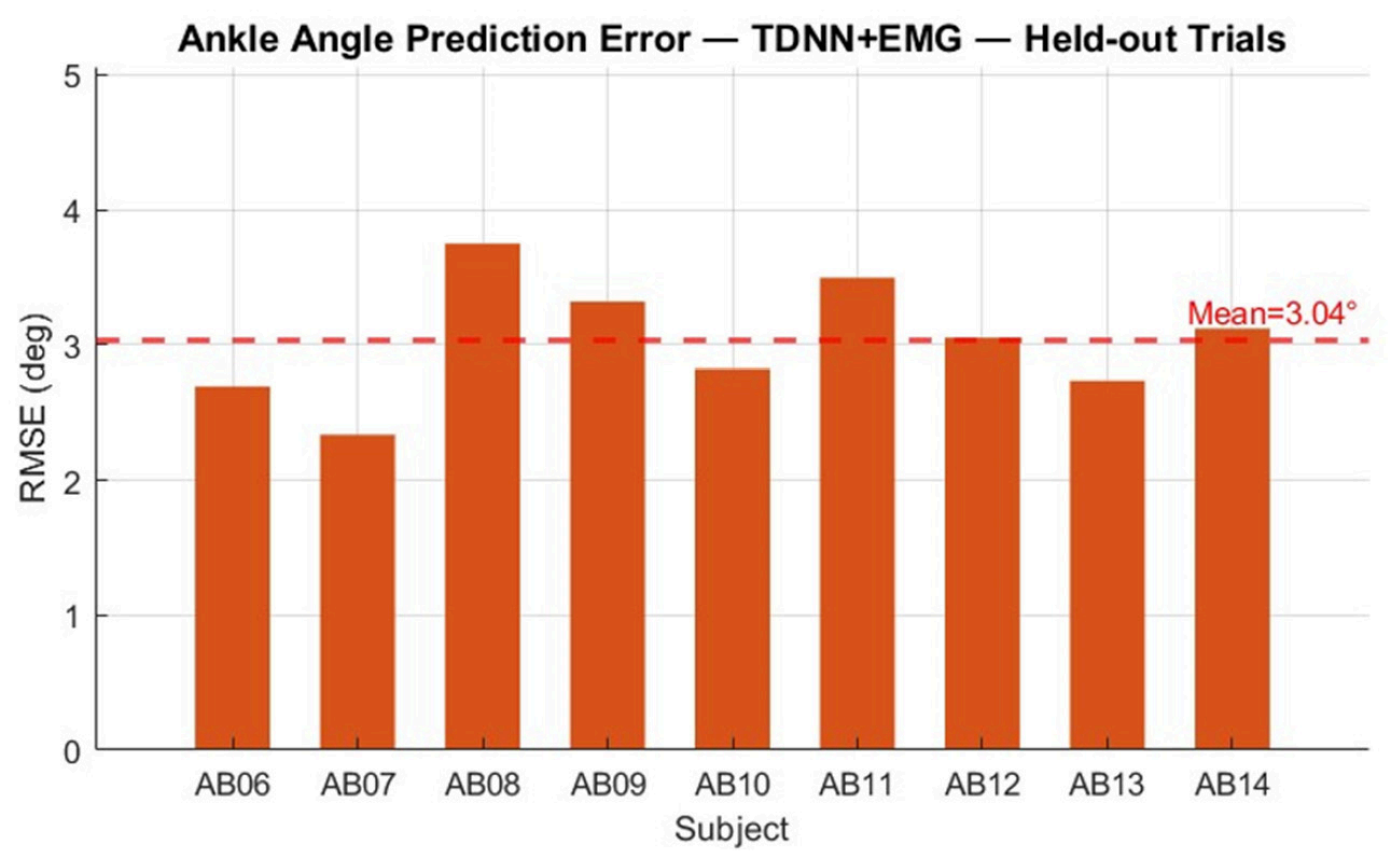
Per-subject RMSE for the TDNN-Kin-EMG model on the held-out trials. The dashed line indicates the across-subject mean RMSE of 3.04°.

The distribution of prediction errors across the nine participants is shown in Figure 9 and Figure 10. For the TDNN-Kin model, the RMSE ranged from 2.61° for AB07 to 4.47° for AB09, with a mean of $3.52\pm 0.55^{\circ }$ (Figure 9). In comparison, the TDNN-Kin-EMG model produced RMSE values ranging from 2.34° for AB07 to 3.75° for AB08, with a lower mean of $3.04\pm 0.44^{\circ }$ (Figure 9).

The inclusion of sEMG reduced both the mean RMSE and its between-subject variability. In particular, the maximum observed RMSE decreased from 4.47° with TDNN-Kin to 3.75° with TDNN-Kin-EMG. As reported in Table 2, the addition of sEMG reduced RMSE for eight of the nine participants, although the magnitude of improvement varied between subjects.

### 4.5. Model Comparison

**Figure 11:**
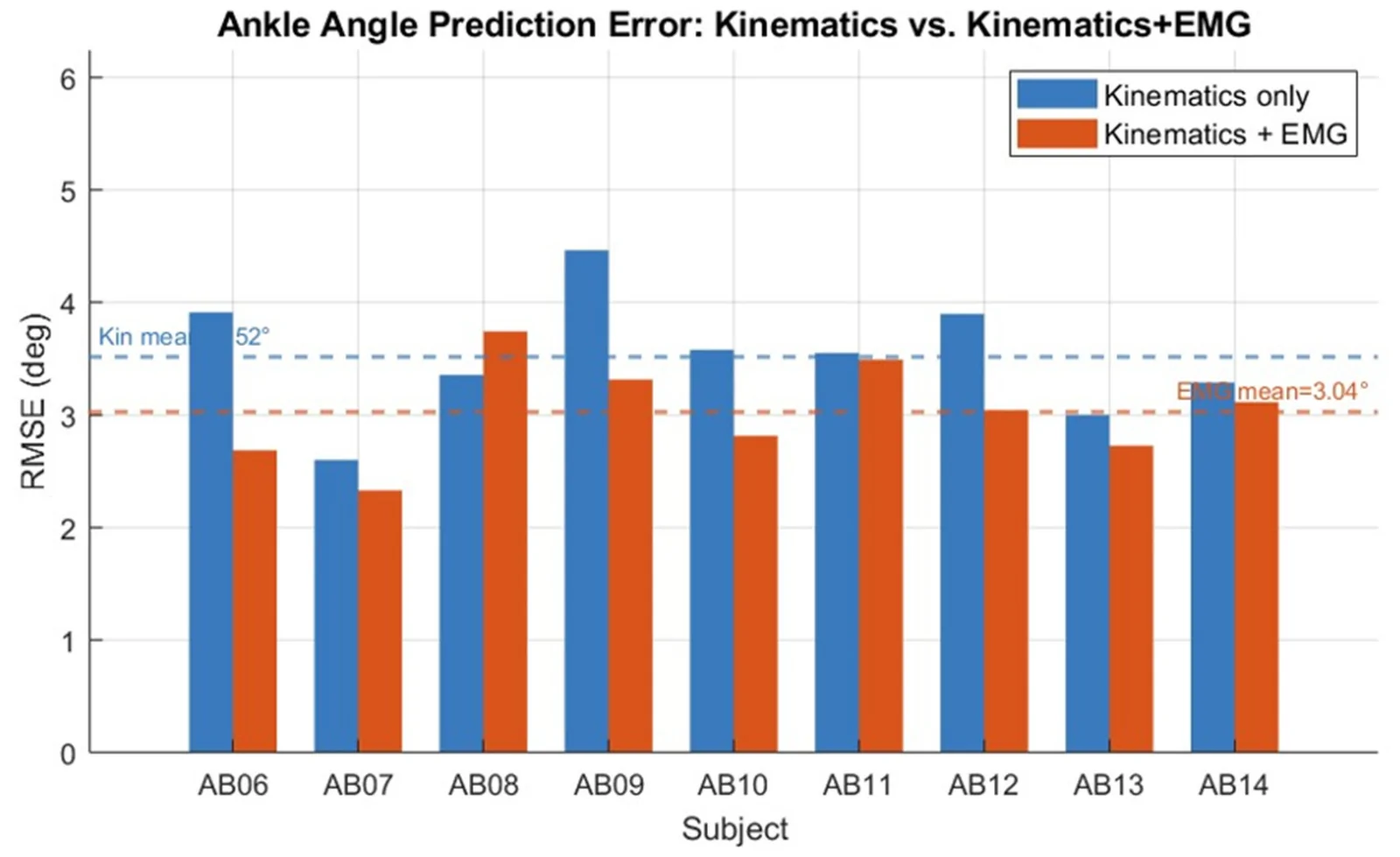
Subject-wise comparison of ankle-angle prediction RMSE for the TDNN-Kin and TDNN-Kin-EMG models on the held-out trials. Horizontal dashed lines indicate the corresponding across-subject mean RMSE values of 3.52° and 3.04°, respectively.

**Figure 12:**
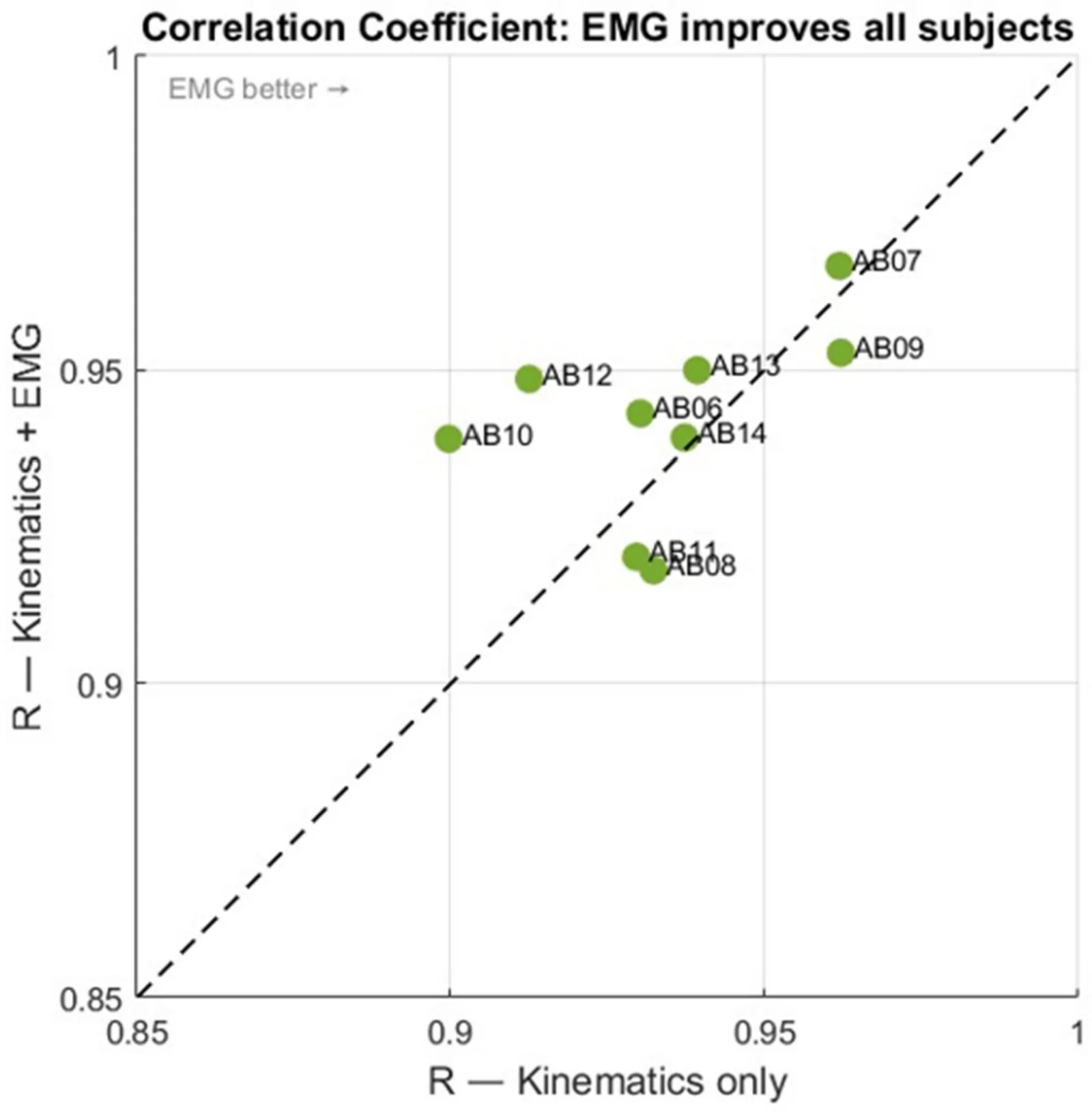
Pearson Correlation Coefficients: TDNN-Kin vs. TDNN-Kin-EMG.

A direct subject-wise comparison of the two TDNN configurations is presented in Figure 11 and Figure 12. As shown in Figure 11, the TDNN-Kin-EMG model achieved a lower RMSE than the TDNN-Kin model for eight of the nine participants. The largest relative reduction was observed for AB06, where the RMSE decreased from 3.92° to 2.70° (31.1%). Substantial reductions were also observed for AB09, AB10, and AB12. In contrast, AB08 showed an increase in RMSE from 3.36° to 3.75° when sEMG was included. Across all participants, the mean RMSE decreased from 3.52° to 3.04°, corresponding to an overall reduction of 13.8%.

The subject-wise comparison of Pearson correlation coefficients is shown in Figure 12. The TDNN-Kin-EMG model produced higher R values for six of the nine participants (AB06, AB07, AB10, AB12, AB13, and AB14), while lower values were obtained for AB08, AB09, and AB11. The largest increase was observed for AB10, where R increased from 0.8997 to 0.9390. Despite the subject-dependent response, the mean correlation coefficient increased from 0.9338 for TDNN-Kin to 0.9419 for TDNN-Kin-EMG.

Taken together, the results show that adding sEMG primarily improved the magnitude of the ankle-angle prediction error, as reflected by the RMSE reduction in eight participants. Improvements in waveform correlation were less consistent across subjects, although the overall mean R remained higher for the TDNN-Kin-EMG configuration.

Although the addition of sEMG improved RMSE for most participants, the magnitude of the improvement varied considerably between individuals.

### 4.6. Predicted Versus Measured Ankle Angles

**Figure 13:**
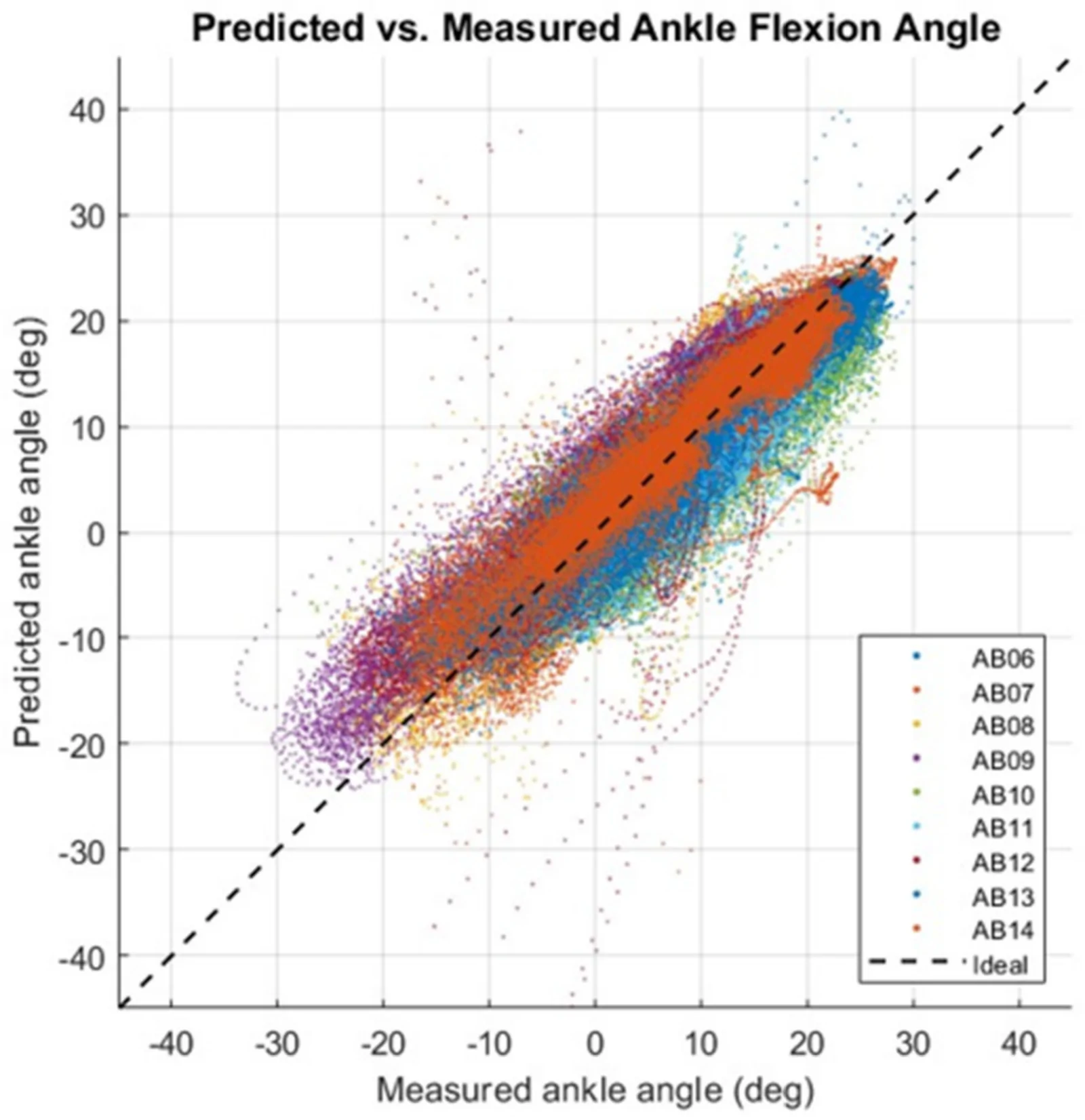
Predicted versus measured ankle angles for the TDNN-Kin model across the held-out trials of all nine participants. The dashed identity line represents perfect agreement between measured and predicted values.

**Figure 14:**
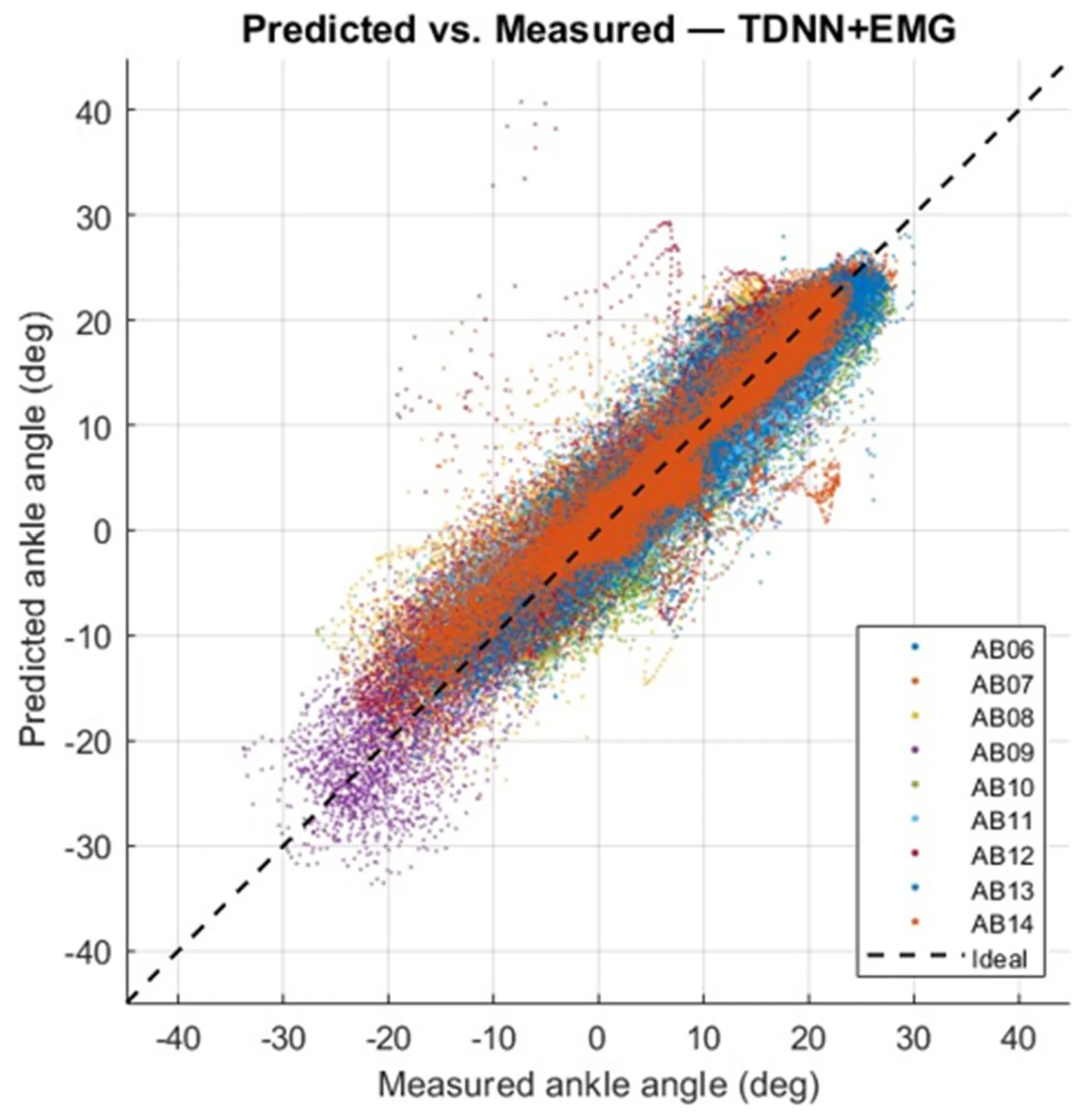
Predicted versus measured ankle angles for the TDNN-Kin-EMG model across the held-out trials of all nine participants. The dashed identity line represents perfect agreement between measured and predicted values.

The relationship between measured and predicted ankle angles across the held-out trials of all nine participants is shown in Figure 13 and Figure 14. For both TDNN configurations, the predicted values were concentrated around the identity line over most of the measured ankle-angle range, consistent with the high correlation coefficients reported in Table 2.

For the TDNN-Kin model (Figure 13), the predicted ankle angles generally followed the measured values, although the dispersion increased in some regions of the angular range and varied between participants. The TDNN-Kin-EMG model showed a similar overall distribution (Figure 14), with predictions remaining closely aligned with the identity line for most observations. The reduced mean RMSE obtained with TDNN-Kin-EMG (3.04° compared with 3.52° for TDNN-Kin) indicates improved overall agreement between the measured and predicted ankle angles when sEMG information was included.

For both models, a small number of observations showed larger deviations from the identity line. These deviations were subject-dependent and were more apparent near portions of the measured angular range with fewer observations. Nevertheless, the overall distributions demonstrate that both TDNN configurations reproduced the measured ankle-angle variation across participants, with lower aggregate prediction error for the TDNN-Kin-EMG model.

### 4.7. Training Error Distribution

**Figure 15:**
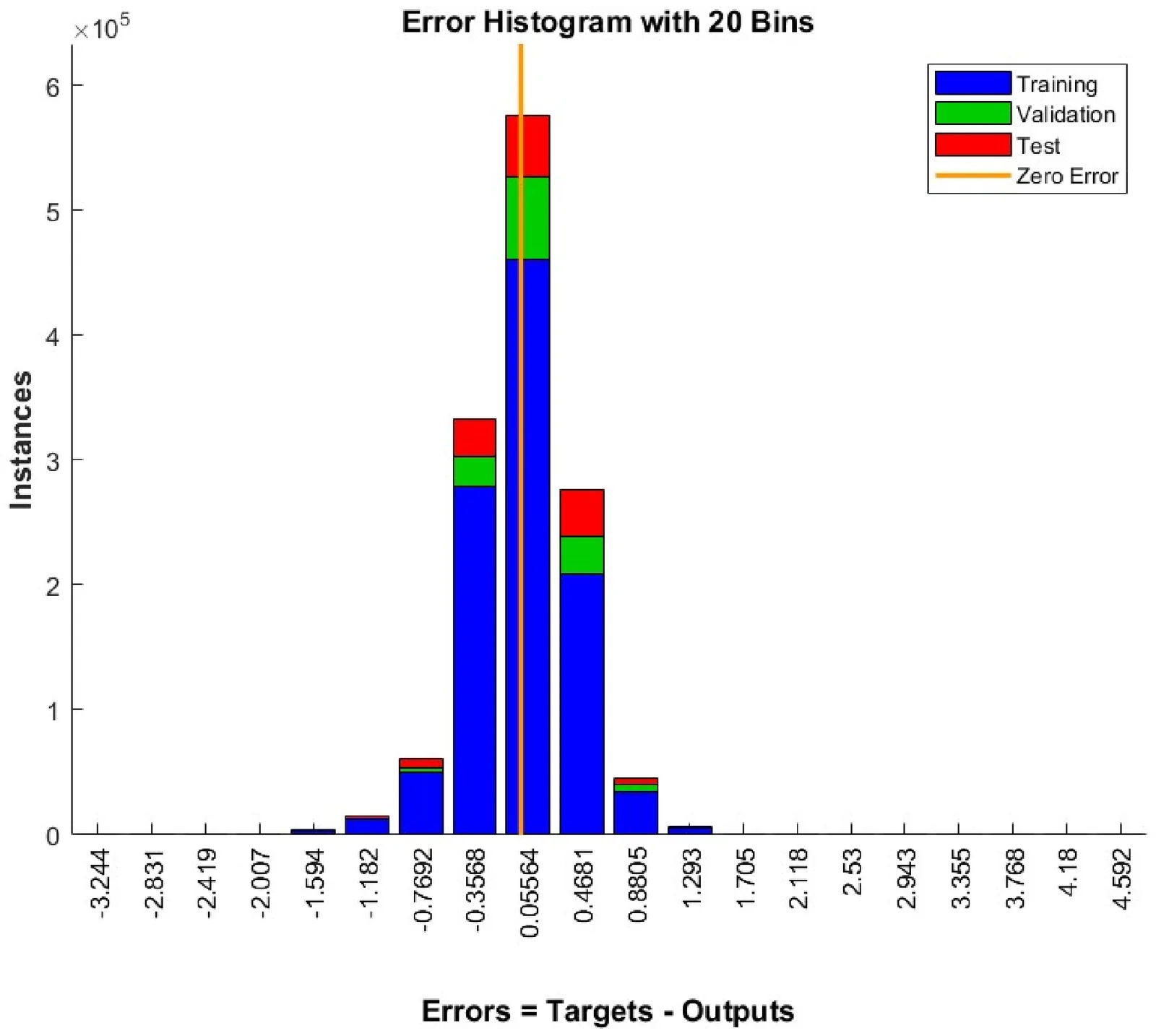
Error histogram for the TDNN-Kin model during model development, showing the distributions for the training, validation, and internal test subsets. The vertical line indicates zero prediction error.

**Figure 16:**
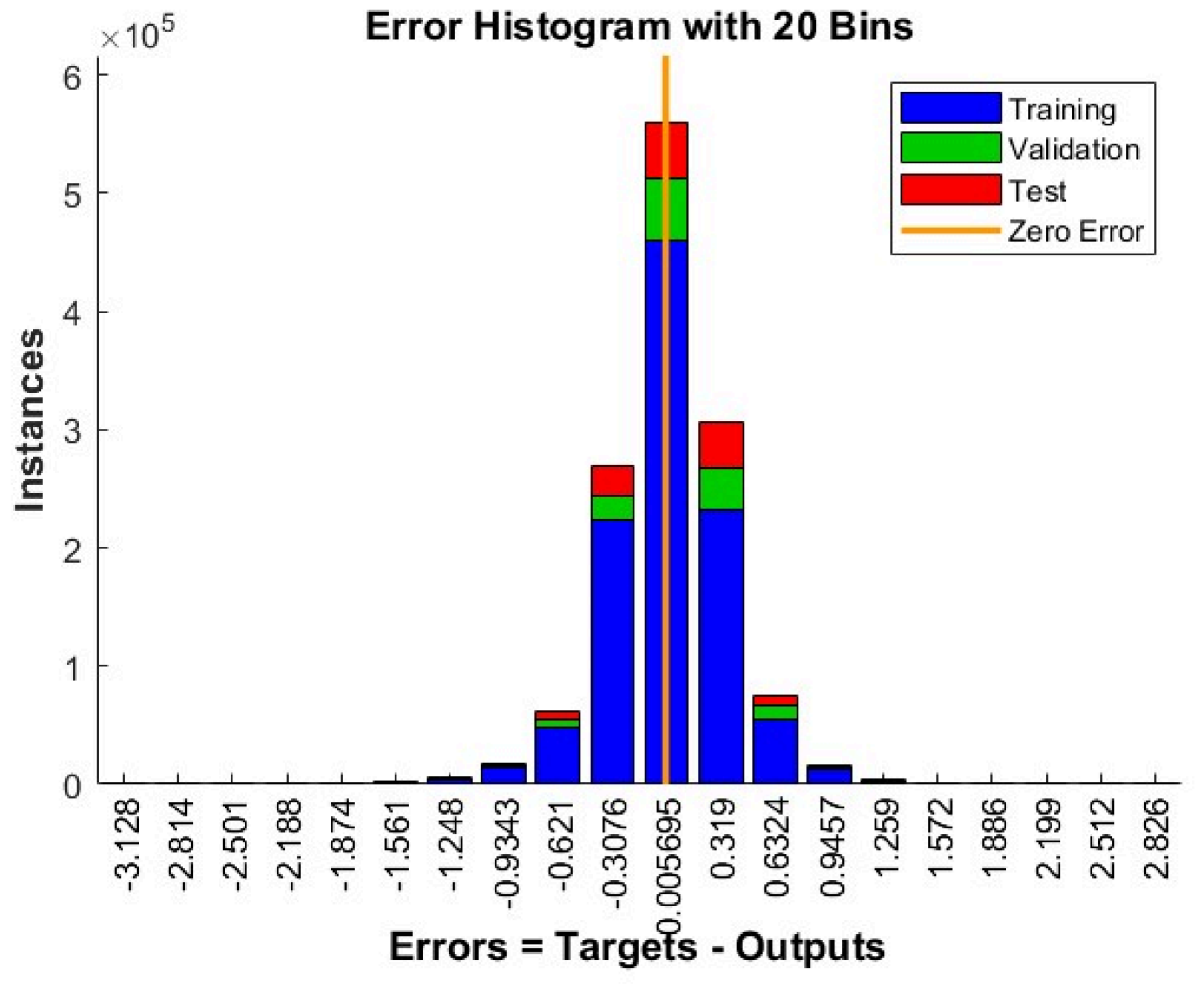
Error histogram for the TDNN-Kin-EMG model during model development, showing the distributions for the training, validation, and internal test subsets. The vertical line indicates zero prediction error.

The error distributions obtained during model development are shown in Figure 15 and Figure 16. For both TDNN configurations, the majority of prediction errors were concentrated around zero, while the frequency of larger positive and negative errors decreased toward the tails of the distributions. Similar patterns were observed across the training, validation, and internal test subsets.

For the TDNN-Kin model (Figure 15), the error distribution was centered close to zero, with most observations concentrated within the central histogram bins. The TDNN-Kin-EMG model exhibited a similarly centered distribution (Figure 16), with a greater concentration of observations near zero and a narrower overall error range. This pattern is consistent with the lower prediction error obtained when the three sEMG channels were included.

The similarity of the training, validation, and internal test error distributions is also consistent with the convergence behavior presented in Figure 5 and Figure 6. These results characterize the error distribution during model development; prediction performance on the independent held-out trials is reported separately in Section 4.2, Section 4.3, Section 4.4, Section 4.5 and Section 4.6.

## 5. Discussion

The present results demonstrate that sagittal-plane ankle motion can be predicted from proximal lower-limb kinematics with relatively low error. Using hip and knee kinematics together with gait-phase information, the TDNN-Kin model achieved a mean RMSE of 3.52 ± 0.55° and a mean correlation coefficient of R = 0.9338 on the independent held-out trials. These findings support the existence of coordinated relationships between proximal-joint motion and ankle kinematics during walking, consistent with previous biomechanical studies [29,30,31].

Adding sEMG information further improved prediction accuracy. The TDNN-Kin-EMG model reduced the mean RMSE to 3.04 ± 0.44°, corresponding to a 13.8% reduction, while the mean correlation coefficient increased from 0.9338 to 0.9419. RMSE improved in eight of the nine participants, although the improvement varied between individuals. This finding suggests that sEMG provides complementary information beyond joint kinematics alone, in agreement with previous studies using myoelectric signals for ankle-motion prediction and adaptive control [28,33].

The variability observed across participants indicates that the contribution of sEMG is subject-dependent. Surface EMG is influenced by electrode placement, skin conditions, and individual muscle activation patterns, which may partly explain why improvements were not consistent for every participant. In the present study, these factors were not evaluated separately and therefore cannot be quantified.

The proposed TDNN uses a 20-sample (100 ms) temporal window, allowing recent kinematic and muscle-activation history to be incorporated without recurrent feedback. This feedforward structure is computationally simple and avoids error accumulation associated with recurrent architectures, making it suitable for future implementation in embedded prosthesis controllers.

The proposed prediction framework is intended to provide reference ankle trajectories for the active ankle prosthesis described in Section 2. However, the present study evaluated offline prediction only. The TDNN output has not yet been integrated into the physical prosthesis or validated under real-time closed-loop control. Therefore, the reported results demonstrate the feasibility of the prediction framework rather than the performance of a complete prosthesis controller.

Several limitations should be acknowledged. First, the analysis was limited to nine able-bodied participants performing treadmill walking. Second, although one trial from each participant was reserved for independent testing, all participants contributed to model development; therefore, the results represent generalization to unseen trials rather than to unseen individuals. Third, only three sEMG channels were evaluated, and alternative muscle combinations were not investigated.

Future work will include validation using data from individuals with lower-limb amputation, evaluation under different locomotor conditions, including ramps, stairs, and walking-speed transitions, and subject-independent testing. The trained TDNN will also be integrated into the embedded control system of the active ankle prosthesis for real-time experimental validation.

## 6. Conclusions

This study presented a TDNN-based framework for predicting sagittal-plane ankle motion from proximal lower-limb information, with the long-term objective of generating reference trajectories for active ankle prostheses. Two TDNN configurations were evaluated: a kinematics-based model using hip and knee kinematics together with gait-phase information, and an extended model incorporating three sEMG channels.

Evaluation on independently held-out treadmill trials from nine able-bodied participants demonstrated that ankle motion can be predicted accurately from proximal-joint information. The TDNN-Kin model achieved a mean RMSE of 3.52 ± 0.55°, which decreased to 3.04 ± 0.44° with the inclusion of sEMG, corresponding to a 13.8% reduction. RMSE improved in eight of the nine participants, indicating that sEMG provides complementary information for ankle-angle prediction, although its benefit is subject-dependent.

Overall, the proposed framework demonstrates the feasibility of using a feedforward TDNN for offline ankle-angle prediction from wearable kinematic and physiological signals. However, the present results are limited to offline evaluation using treadmill-walking data from able-bodied participants and should not be interpreted as validation of a real-time prosthesis controller. Future work will focus on subject-independent evaluation, validation using data from individuals with lower-limb amputation, and real-time implementation of the prediction framework on the developed active ankle-foot prosthesis.

## Figures and Tables

**Table 1:** Demographic characteristics of the participants included in the study.

Subject	Age (yr)	Sex	Height (cm)	Mass (kg)	Leg Length (cm)	Training/Development Trials
AB06	23	M	180.3	79.5	94.0	14
AB07	24	M	175.3	72.6	91.4	14
AB08	22	M	177.8	77.1	93.3	14
AB09	26	F	162.6	62.1	84.5	14
AB10	24	M	180.3	88.5	95.3	14
AB11	25	M	182.9	79.8	96.5	14
AB12	22	F	162.6	56.7	83.8	14
AB13	27	M	177.8	81.6	93.5	14
AB14	24	M	175.3	75.7	92.1	14
Mean ± SD	24.1 ± 1.7	7M/2F	175.0 ± 7.4	74.8 ± 9.9	91.6 ± 4.5	9 × 14 = 126

Note: Participant information is reproduced from Camargo et al. [12]. One treadmill_07_01 trial from each participant was reserved as an independent held-out test trial and was excluded from model development. The remaining 14 trials per participant (126 trials in total) were used for model development.

**Table 2:** Held-out ankle-angle prediction performance for the TDNN-Kin and TDNN-Kin-EMG models.

Subject	RMSE (°), TDNN-Kin	R, TDNN-Kin	RMSE (°), TDNN-Kin-EMG	R, TDNN-Kin-EMG	ΔRMSE	ΔR
AB06	3.92	0.9302	2.70	0.9430	−31.1%	+0.013
AB07	2.61	0.9619	2.34	0.9665	−10.3%	+0.005
AB08	3.36	0.9323	3.75	0.9181	+11.6%	−0.014
AB09	4.47	0.9621	3.32	0.9527	−25.7%	−0.009
AB10	3.58	0.8997	2.82	0.9390	−21.2%	+0.039
AB11	3.56	0.9295	3.50	0.9202	−1.7%	−0.009
AB12	3.90	0.9124	3.05	0.9485	−21.8%	+0.036
AB13	3.01	0.9392	2.74	0.9499	−9.0%	+0.011
AB14	3.29	0.9372	3.12	0.9392	−5.2%	+0.002
Mean ± SD	3.52 ± 0.55	0.9338	3.04 ± 0.44	0.9419	−13.8%	+0.008

## Data Availability

The data presented in this study are available on request from the corresponding author.
